# CAU-Net: A Deep Learning Method for Deep Gray Matter Nuclei Segmentation

**DOI:** 10.3389/fnins.2022.918623

**Published:** 2022-06-02

**Authors:** Chao Chai, Mengran Wu, Huiying Wang, Yue Cheng, Shengtong Zhang, Kun Zhang, Wen Shen, Zhiyang Liu, Shuang Xia

**Affiliations:** ^1^Department of Radiology, Tianjin Institute of Imaging Medicine, Tianjin First Central Hospital, School of Medicine, Nankai University, Tianjin, China; ^2^College of Electronic Information and Optical Engineering, Nankai University, Tianjin, China; ^3^School of Medicine, Nankai University, Tianjin, China; ^4^Tianjin Key Laboratory of Optoelectronic Sensor and Sensing Network Technology, Tianjin, China

**Keywords:** convolutional neural network (CNN), deep learning, medical image segmentation, gray matter nuclei, quantitative susceptibility mapping, strategically acquired gradient echo (STAGE) imaging

## Abstract

The abnormal iron deposition of the deep gray matter nuclei is related to many neurological diseases. With the quantitative susceptibility mapping (QSM) technique, it is possible to quantitatively measure the brain iron content *in vivo*. To assess the magnetic susceptibility of the deep gray matter nuclei in the QSM, it is mandatory to segment the nuclei of interest first, and many automatic methods have been proposed in the literature. This study proposed a contrast attention U-Net for nuclei segmentation and evaluated its performance on two datasets acquired using different sequences with different parameters from different MRI devices. Experimental results revealed that our proposed method was superior on both datasets over other commonly adopted network structures. The impacts of training and inference strategies were also discussed, which showed that adopting test time augmentation during the inference stage can impose an obvious improvement. At the training stage, our results indicated that sufficient data augmentation, deep supervision, and nonuniform patch sampling contributed significantly to improving the segmentation accuracy, which indicated that appropriate choices of training and inference strategies were at least as important as designing more advanced network structures.

## Introduction

In the last decade, the advent of the quantitative susceptibility mapping (QSM) technique can achieve the quantitative measurement of brain iron content *in vivo* (Langkammer et al., 2010; Liu et al., 2015, 2017). QSM employed the magnetic susceptibility of tissue as the inherent physical magnetic resonance imaging (MRI) parameter, which indicated how the local magnetic field in tissues changes when an external magnetic field is applied (Li et al., 2019). Magnetic susceptibility of tissue can provide unique information of tissue iron composition (Li et al., 2019). Compared with other iron-sensitive techniques, including the transverse relaxation rates (R_2_, R_2_*, and R_2_’), field-dependent rate increase, phase information from susceptibility-weighted imaging (SWI), and magnetic field correlation imaging, QSM can overcome the limitations of these techniques, such as the relatively low accuracy of R_2_* due to other confounding factors (water content and calcium), geometry- and orientation-dependence of phase images, and low sensitivity to small changes in brain iron (Stankiewicz et al., 2007; Bilgic et al., 2012; Deistung et al., 2013; Chai et al., 2019). QSM was more accurate in measuring the iron content and strongly correlated with the iron concentration of postmortem brain tissues (Langkammer et al., 2010).

The quantitative measurement of brain iron content using QSM has brought into focus the role of iron in the brain development, physical function modulation, and aging (Salami et al., 2018; Peterson et al., 2019), as well as in various neurological diseases, including Alzheimer’s disease, Parkinson’s disease, multiple sclerosis, metabolic diseases (hepatic encephalopathy and renal encephalopathy), sleep disorders, hematological system diseases, and cerebrovascular diseases (Chai et al., 2015a; Xia et al., 2015; Miao et al., 2018; Chai et al., 2019; Valdés Hernández et al., 2019; Pudlac et al., 2020; Cogswell et al., 2021; Thomas et al., 2021; Zhang et al., 2021; Galea et al., 2022). As iron has been proned to accumulate in the gray matter nuclei in normal people and all these neurological diseases have abnormal iron deposition in the gray matter nuclei, the gray matter nuclei are the critical target structures to explore the abnormal iron deposition. Previous studies have found that routine structural MR images such as T_1_-weighted images could hardly show iron-rich gray matter nuclei clearly, such as substantia nigra (SN), red nucleus (RN), and dentate nucleus (DN; Beliveau et al., 2021). Therefore, these nuclei were not found in the most popular brain atlas, including FreeSurfer, FMRIB Software Library (FSL), and Statistical Parametric Mapping (SPM). Most segmentation tools cannot extract these nuclei (Beliveau et al., 2021). However, all the gray matter nuclei, including SN, RN, and DN, showed the obvious contrast (high signal) relative to the surrounding brain tissues in the QSM images because QSM was very sensitive to the iron, even when the amount was small and QSM can also enhance the iron-related contrast (Beliveau et al., 2021). The apparent contrast can help to identify the gray matter nuclei clearly and accurately. The measurement of iron content needs to manually outline the volumes of interest (VOIs) of the gray matter nuclei, which heavily depend on the operator’s experience and cause some bias (Chai et al., 2022). The manual drawing of VOIs was also a tedious task and consumed an amount of time, which limited the wide application beyond research interest. To date, one study has used the SWI as the target modality because SWI can provide the enhanced contrast to visualize the gray matter nuclei compared to the other iron-sensitive modalities besides QSM and SWI also has a wide range of clinical applications (Beliveau et al., 2021). However, it was not far from enough to visualize and segment the nuclei using SWI, and the quantitative measurement of iron content was also a very critical step for the clinical evaluation of abnormal iron deposition for the diagnosis of neurological diseases. Therefore, QSM as the target modality can provide the enhanced contrast as good as SWI and directly quantitatively provide the information about iron content (Liu et al., 2015).

Deep learning has recently been successfully applied in biomedical image segmentation tasks (Minaee et al., 2021). It has been shown that, in many medical image segmentation tasks, such as tumor segmentation (Menze et al., 2015; Chang et al., 2018), stroke lesion segmentation (Maier et al., 2017; Liu et al., 2018), and organ segmentation (Gibson et al., 2018), deep learning methods were able to significantly exceed the conventional atlas-based methods. Most deep-learning-based medical image segmentation tasks adopted the U-Net (Ronneberger et al., 2015) or its variants (Cicek et al., 2016; Chang et al., 2018; Liu et al., 2018; Meng et al., 2018; Wang et al., 2020). By introducing dense skip connections between the encoder and decoder layers, U-Net like structures were able to effectively fuse the spatial and semantic information even when the training set was small. To further improve the segmentation accuracy of U-Net, some modifications at the encoder part or at the skip connections were proposed in the literature. The modications at the encoder mainly focused on making the encoders wider (Chen et al., 2019; Wang et al., 2019; Ibtehaz and Rahman, 2020), so as to enrich the feature maps from multiple fields of view. At the skip connections, the modifications were applied by incorporating various attention mechanisms to guide the decoder to utilize the most essential features (Oktay et al., 2018; Guo et al., 2021).

When applied to the brain gray matter nuclei segmentation task, deep learning methods have also been more robust and accurate than the atlas-based methods (Guan et al., 2021; Chai et al., 2022). For instance, Chai et al. (2022) proposed a double-branch U-Net structure for gray matter nuclei segmentation in the QSM images, which incorporated the local feature maps from image patches with the original resolutions and the global feature maps from down-sampled image patches and presented high accuracy in nuclei segmentation with a light-weighted neural network. Guan et al. (2021) also developed a segmentation method known as DeepQSMSeg to segment five pairs of nuclei, including CN, PUT, GP, SN, and RN in the QSM images, which incorporated the spatial-wise and channel-wise attention mechanism into the U-Net architecture.

Most deep-learning methods mainly focused on proposing novel network architectures, and most of them were developed based on U-Net. The training strategies, however, were not emphasized. In this study, we attempted to emphasize not only the network structures, but also the importance in fine tuning the networks with appropriate training and inference strategies. In particular, we adopted a minor modification in the U-Net by introducing contrast attention (CA) modules at the skip connections and attempted to improve the segmentation accuracy without introducing additional network parameters. Experiments were conducted on two different datasets (Datasets I and II) with QSM acquired using different MRI sequences with different imaging parameters from different MRI devices. Dataset I was randomly split as a training set with 42 subjects and a test set with 20 subjects. The network was trained on the training set and evaluated on the test set and Dataset II. Experimental results revealed that on both datasets, the proposed method was able to overperform the other popular U-Net-shaped structures, including 3D U-Net (Cicek et al., 2016), Attention U-Net (Oktay et al., 2018), and DeepQSMSeg (Guan et al., 2021), which highlighted the ability of generalization of our proposed method. The effects of various training strategies were also discussed, which implied that data augmentation, deep supervision, and nonuniform patch sampling were beneficial for improving the segmentation accuracy.

## Materials and Methods

### Datasets

This prospective study was approved by the Tianjin First Central Hospital Review Board and Ethics Committee. The informed consent of all subjects was obtained before the MRI examination. Our study included two datasets acquired using different MRI sequences from different MRI devices, Dataset I with sixty-two healthy subjects (age range 22–60 years, mean age 37.34 ± 11.32 years; male 24 and female 38) and Dataset II with twenty-six healthy subjects (age range 54–72 years, mean age 62.44 ± 4.35 years; male 18 and female 9). All were enrolled from Tianjin First Central Hospital staff or community members by advertisement. The inclusion criteria were as follows: (1) the age of the subjects was 18 years or older; (2) the subjects had no MRI contraindications, including metal implant, pacemaker, or claustrophobia; (3) the subjects had no history of central nervous system diseases, including the cerebral infarction, cerebral hemorrhage, cerebral tumor, traumatic cerebral injury, or contusion, which might affect the segmentation of the cerebral structures. The exclusion criteria were as follows: (1) the subjects cannot finish the MRI scanning and acquire the available SWI images and 3D T_1_-weighted images; (2) the subjects had the congenital abnormalities and above central nervous system diseases, which might affect the segmentation of the cerebral structures; (3) the quality of MRI images was not good for the post process and analysis.

Dataset I was randomly split into the training set and test set, with 42 (age range 22–55 years, mean age 36.6 ± 10.94 years; male 15 and female 27) and 20 subjects (age range 25–60 years, mean age 38.9 ± 12.22 years; male 9 and female 11), respectively. The training set was used to train the neural networks, while the test set was used to evaluate the performance. All subjects in Dataset II were used for evaluation.

MRI data of Dataset I included SW images and 3D T_1_W images and were collected using a 3.0 T MRI scanner (Magnetom TIM TRIO scanner, Siemens Healthineers, Erlangen, Germany) equipped with an 8-channel phased-array head coil. The acquisition parameters of Dataset I were listed as follows: (1) the parameters of SWI: TR (time repetition)/TE (time echo) = 27/20 ms, number of slices = 56, FoV = 230 mm × 200 mm, voxel resolution = 0.5 mm × 0.5 mm × 2 mm, corresponding matrix sizes = 336 × 448 × 56, receiver bandwidth = 120 Hz/pixel, flip angle = 15°, and acquisition time = 334 s; (2) the parameters of 3D T_1_WI: TR/TE = 1,900/2.52 ms, TI (time inversion) = 900 ms, number of slices = 176, FoV = 250 × 250 mm^2^, voxel size = 1.0 mm × 1.0 mm × 1.0 mm, corresponding matrix sizes = 256 × 256 × 176, flip angle = 9°, and acquisition time = 258 s. MRI data of Dataset II were collected using another 3.0T MRI scanner (MAGNETOM Prisma, Siemens Healthcare, Erlangen, Germany) equipped with a 20-channel phased-array head coil.

The subjects of Dataset II had strategically acquired the gradient echo (STAGE)-MR angiography and venography (MRAV) sequence instead of the SWI sequence and also 3D T_1_WI. The STAGE-MRAV sequence is a multi-parametric MRI sequence, which can be post-processed to acquire the QSM images directly. The acquisition parameters of Dataset II were listed as follows: (1) the parameters of STAGE sequence: TR/TE = 20/(2.5, 12.5) ms, matrix sizes = 384 × 288, flip angle = 12°, number of slices = 64, slice thickness = 2 mm, in-plane spatial resolution = 0.67 mm × 0.67 mm, FoV = 256 mm × 192 mm, receiver bandwidth/pixel = 240 Hz/pixel, and total acquisition time = 368 s; (2) the parameters of 3D T_1_WI: TR/TE = 2,000/2.98 ms, TI = 900 ms, number of slices = 176, FoV = 256 mm × 248 mm, voxel size = 1.0 mm × 1.0 mm × 1.0 mm, corresponding matrix sizes = 256 × 248 × 176, flip angle = 9°, and acquisition time = 269 s.

Considering that the SWI and QSM images of STAGE-MRAV and 3D T_1_WI were acquired using different parameters and different FoVs, we first registered the T_1_WI images and the SWI images or QSM images of STAGE-MRAV using rigid affine transformation with mutual information as the criterion, and then resampled the T_1_WI images using linear interpolation, so that the T_1_WI image and its corresponding SWI image or QSM images of STAGE-MRAV were with the same spatial resolutions and matrix sizes.

The QSM images were reconstructed from the phase and magnitude images of SWI by employing the SMART software (Susceptibility Mapping and Phase Artifacts Removal Toolbox, Detroit, MI. The QSM images from the STAGE-MRAV sequence were acquired using the STAGE software (SpinTech Inc., MI, United States). The postprocessing steps of reconstruction of QSM have been reported in several studies (Chai et al., 2015b, 2022; Tang et al., 2020; Zhang et al., 2021). First, the elimination of the skull and other regions with low signals was performed using the Brain Extraction Tool (BET) in the FMRIB Software Library (FSL; Smith, 2002). Second, excluding the phase wraps in the original phase images was performed using a 3D best-path algorithm (Abdul-Rahman et al., 2007). Third, the elimination of the background phase information was performed using a sophisticated harmonic artifact reduction for the phase data (SHARP) algorithm (Schweser et al., 2011). Finally, the reconstruction of QSM images was performed using the truncated k-space division algorithm with a k-space threshold of 0.1 (Haacke et al., 2010).

### Manual Annotation

**FIGURE 1:**
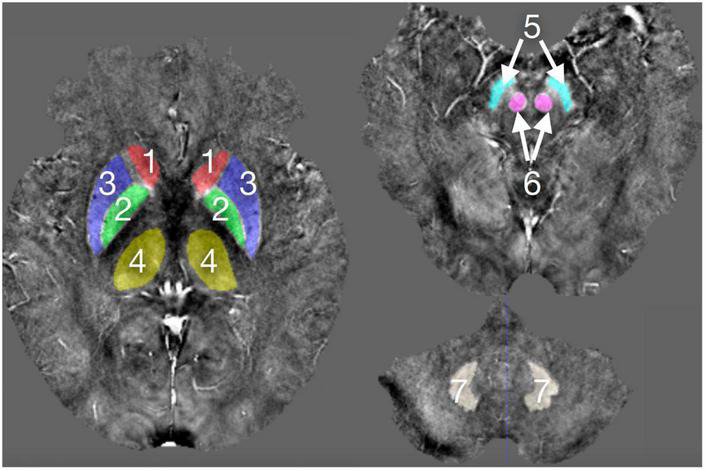
The deep gray matter nuclei of interest outlined in the QSM images.1, CN; 2, GP; 3, PUT, 4, THA; 5, SN; 6, RN; and 7, DN.

The drawing of gray matter nuclei’s volume of interest (VOI) in the QSM images was performed using the SPIN software (Signal Processing in Nuclear Magnetic Resonance, Detroit, MI, United States). The gray matter nuclei in our study included the bilateral caudate nuclei (CN), globus pallidus (GP), putamen (PUT), thalamus (THA), red nuclei (RN), substantia nigra (SN), and dentate nuclei (DN), as shown in Figure 1. These nuclei showed a high signal in the QSM images. Considering the personal difference in the shape and size of the nuclei in different people and in order to assure that the susceptibility values were assessed as accurately as possible for each subject, the VOIs were outlined manually on the contiguous slices of gray matter nuclei to include the whole volume of each nucleus by two well-trained neuroradiologists (C.C. and H.Y.W.) with 11 and 6 years of experience in neuroradiology who were blinded to the clinical and epidemiological information. When drawing the VOIs of the nuclei, we also magnified the images to obtain the more precise margin of nuclei. The topmost and lowermost slices of nuclei were excluded to eliminate the influence of edge partial volume effects. The susceptibility values of gray matter nuclei were presented as mean values and standard deviation.

### Proposed Method

**FIGURE 2:**
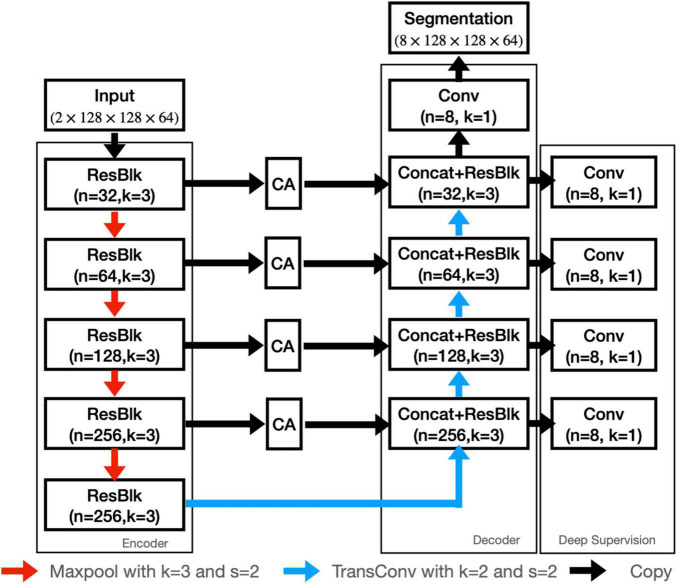
The architecture of our proposed CAU-NET. The red arrows denote maxpooling, and the blue arrows denote the transposed convolution. The black arrows denote the copying of feature maps. “Concat” denotes the channel-wise concatenation. “ResBlk” denotes the residual block, whose structure is shown in Figure 3.

**FIGURE 3:**
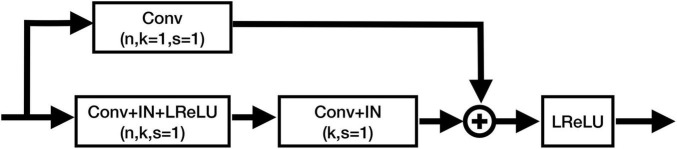
Structure of the residual blocks in CAU-Net. Conv, ConvTrans, IN, and LReLU denote the convolution layer, transposed convolution layer, instance normalization, and Leaky ReLU activation function, respectively. “+” denotes the element-wise addition.

In this study, we employed both T1WI and QSM for nuclei segmentation, so as to utilize the high structural contrast of T1WI and the enhanced iron-related contrast of QSM. To better segment the nuclei, a contrast-attention U-Net (CAU-Net) was proposed for nuclei segmentation. In the classical U-Net, skip connections were employed to fuse the feature maps hierarchically with the decoder feature maps. In our proposed network, the CA module was added at the skip connections to encourage the network to extract the most prominent features and pass them to the decoder. As shown in Figure 2, the proposed CAU-Net employed a U-Net like structure in general, but made several significant modifications. The detailed hyperparameters, such as the numbers of filters and kernel sizes, can be found in Figures 2, 3.

#### Contrast Attention

U-Net is the most successful network architecture in medical image segmentation, which fuses high-level and low-level features by skipping connections to obtain rich contextual information and precise location information.

Basically, to obtain accurate segmentation results, the network should be able to utilize both semantic and spatial information. In the encoder layers of a U-Net, the semantic information is extracted by many consecutive convolution layers, making it necessary to down-sample the feature maps to enlarge the FoVs of the convolution layers. In the decoder part, to recover the spatial information and generate accurate segmentation, it has to utilize both the semantic information from the deepest layer of the encoder and the spatial information from the shallower layers of the encoder. To generate a fine segmentation map, contour information and local details of images are meaningful for semantic segmentation. For instance, high-pass filters, such as Sobel and Laplacian operators, are widely used to extract the image’s contour in image signal processing. Therefore, we assume that it is more important to pass the contour information to the decoder layers, instead of directly passing all output feature maps of the encoder layers to the decoder.

**FIGURE 4:**
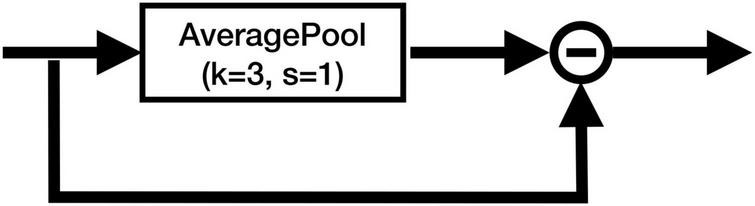
Structure of the CA module. “−” denotes the element-wise subtraction.

To cope with this problem, we added the CA at the skip connections of the U-Net, which can remove the identical information and extract the local differential information from the feature maps. Figure 4 shows the structure of the CA module. The CA module does not include any parameter, and it is simply calculated as follows:


(1) $$Y=X-Avg_{3}\left(X\right),$$

where *Avg*_3_(*X*) denotes the output of the average pooling layer with kernel size 3 and stride 1. It can be easily seen that the CA module works as a high-pass filter, which captures the local differential information and filters out the identical information from each feature map. It can also be interpreted as an implicit edge attention module, making the model better distinguish the edges of different tissues.

#### Training Strategy

Before training, both the T_1_WI and the QSM images were normalized to zero mean and unit variance. The mean and variance values were calculated on all foreground regions of the training set. The T_1_WI and corresponding QSM images were then concatenated to a dual-channel 3D image.

Due to limited GPU memory, cutting the whole volume into volumetric patches was necessary and commonly used in training 3D CNN segmentation networks. In our method, the whole volume was split into multiple patches with the size 128 × 128 × 32. The patches were randomly sampled while ensuring that at least 2/3 of the patches were centered at the foreground voxels.

In our study, the training dataset size was significantly limited. Deep supervision was adopted to train the millions of network parameters and force the convolution layers to efficiently extract valuable features. In particular, a convolution layer with softmax activation was used at each stage of the decoder to generate a segmentation map, as shown in Figure 2. The deep supervision outputs were then up-sampled to the original size and the losses were computed. All deep supervision losses were summed up with the loss at the final output with equal weights, and the sum loss was used to update the network parameters. We used the same loss function at the deep supervision outputs and the final output, which was the sum of Dice loss and the cross entropy loss given as follows:


(2) $$L\left(y,\hat{y}\right)=-\sum_{k=0}^{7}\sum_{i}y_{i,k}\;\log{\hat{y}}_{i,k}-\sum_{k=1}^{7}\frac{2\sum_{i}y_{i,k}\cdot {\hat{y}}_{i,k}}{\sum_{i}y_{i,k}+\sum_{i}{\hat{y}}_{i,k}},$$

where *y*_*i*,*k*_ ∈ {0, 1} denotes whether the *i*-th voxel was classified as the *k*-th class or not, and ${\hat{y}}_{i,k}$ denotes the value of the *i*-th voxel at the *k*-th channel of the network output. It is noted that we only computed the Dice loss of the foreground voxels because the numbers of the foreground and the background voxels were significantly imbalanced.

Sufficient data augmentation is another appropriate technique in dealing with the small training dataset. In our method, we used random zooming, random rotation within the range [−30, 30], random flipping, and random Gaussian smoothing to improve the data diversity. The detailed parameters of data augmentations are summarized in Table 1.

**TABLE 1:** Data augmentation methods adopted in our proposed method.

Data augmentation method	Probability	Parameter
Random flipping	0.5	Along with X, Y, and Z axes
Random zooming	0.2	Range: 0.7–1.3
Random rotation	0.2	Range: [−30,30]
Gaussian smoothing	0.1	σ=0.125
Random intensity rescale	0.2	Range: [0.9,1.1]

The network parameters were initialized as suggested by He et al. (2015). Stochastic gradient descent (SGD) with Nestrov trick was adopted as the optimizer. The momentum was set to be 0.99, and the initial learning rate was set to be 0.01. To train the network parameters sufficiently, we trained the network for a sufficient number of updates. We defined an epoch as 250 batch iterations and trained the network for 500 epochs. The learning rate was adjusted after each epoch, and reduced in a polynomial way.

#### Inference Strategy

During inference, the patches of matrix size 128 × 128 × 32 were extracted from the image with the overlapping rate of 0.5. The whole segmentation map was constructed by combining the segmentations of all patches. Test time augmentation (TTA) was also adopted to further improve the segmentation accuracy. The augmentation included four procedures, namely, augmentation, prediction, disaugmentation, and merging. During the inference, to avoid introducing errors on the segmentation maps due to interpolation, we only used the augmentation methods without requiring interpolation. In particular, we adopted mirroring along all 3 axes and rotating ±90° and generated 8 augmented copies of the original image. We predicted on both the original and the augmented images, and then reverted the transformations on the predictions. Finally, we merged the predictions to generate the final prediction. In our study, we used the soft majority voting method to merge the multiple predictions.

### Evaluation Metrics

In this study, we adopted both symmetric and surface distance metrics to evaluate the segmentation performance. In particular, the symmetric metric we adopted was Dice Coefficient (DC), which was defined as


$$DC_{k}=2\times \frac{\left|P_{k}⋂G_{k}\right|}{\left|P_{k}\right|+\left|G_{k}\right|},$$

where *P_k_* and *G_k_* denote the regions identified as the *k*-th class at the prediction and the ground truth, respectively. |⋅| denotes the area. DC measured how similar the two segmentation maps were.

In addition to DC, we further used surface distance metrics, including surface Dice Coefficient (SDC), Hausdorff distance (HD), and average symmetric surface distance (ASSD) to thoroughly evaluate the segmentation accuracy. These metrics were calculated based on the measurement of the surface distances, i.e., the distances between the surface points of the two segmentation volumes. Similar to the DC, the SDC was also defined as follows:


$$SDC_{k}=2\times \frac{\left|A⋂B\right|}{\left|A\right|+\left|B\right|},$$

where *A* and *B* are surface point sets of the prediction and the ground truth volumes, respectively, and the intersection between the two sets was measured with a given tolerance. In our study, we set the tolerance as 1 mm.

The HD measured the maximum distance between two volume surface points, which was defined as follows:


$$HD\left(A,B\right)=\max\left(\max_{a\in A}\min_{b\in B}d\left(a,b\right),\max_{b\in B}\min_{a\in A}d\left(a,b\right)\right).$$

To reduce the influence of some rare outliers, we used the 95% HD, denoted as HD95, which was obtained by measuring the 95th percentile value instead of the maximum value. The ASSD denoted the average distance between the volume surface points averaged over both directions, which was given as follows:


$$ASSD\left(A,B\right)=\frac{1}{2}\left(\frac{\sum_{a\in A}\min_{b\in B}d\left(a,b\right)}{\left|A\right|}+\frac{\sum_{b\in B}\min_{a\in A}d\left(b,a\right)}{\left|B\right|}\right)$$

Both HD95 and ASSD were given in *mm*, and the lower, the better. Unlike DC and SDC, the HD95 and ASSD worked equally well for large and small objects.

## Results

### Comparative Methods

**FIGURE 5:**
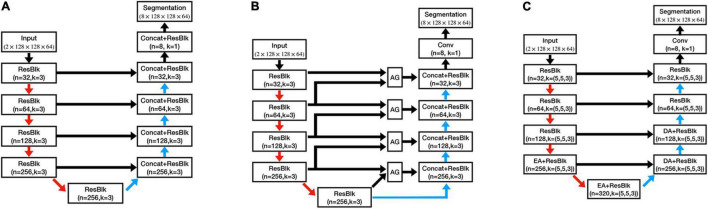
Architectures of the comparative structures. **(A)** U-Net. **(B)** AU-Net. **(C)** DeepQSMSeg. AG denotes the attention gate. EA and DA denote the encoder attention and decoder attention, respectively.

To evaluate the performance of our proposed method, we further trained three other models on the same training set, which were 3D U-Net (Cicek et al., 2016), 3D Attention U-Net (AU-Net;, and DeepQSMSeg (Guan et al., 2021). The network structures can be found in Figure 5, and the detailed structures of the attention modules can be found in their studies.

The U-Net had a similar structure to the CAU-Net, and the only difference was the absence of the CA modules. On the other hand, the AU-Net introduced an additive soft attention mechanism at the attention gates (AGs) at the skip connections of U-Net. The AG fused the feature maps from the current layer and the next lowest layer of the network to generate the attention weights for the most critical positions. DeepQSMSeg was a network structure specifically designed for nuclei segmentation from the QSM. It employed the basic encoder-decoder structure as U-Net, while inserting attention modules between the last two encoder stages and the first two decoder stages to capture the small target structures’ semantic features. In each attention module of DeepQSMSeg, the channel-wise attention and spatial-wise attention were consecutively used to exploit both the channel and spatial relationships, and guide the decoder to generate a finer segmentation.

In our study, as the U-Net and AU-Net were not specifically designed for nuclei segmentation, we used the same training strategy as our proposed one. For DeepQSMSeg, we strictly followed the training protocol introduced by Guan et al. (2021).

### Implementation Setup

The experiments were performed on a workstation with an Intel Core i7-7700K CPU, 64GB RAM, and Nvidia Geforce GTX 1080Ti GPU with 11GB memory. The workstation operated on Linux (Ubuntu 18.04 LTS) with CUDA 11.1. The networks were implemented on PyTorch (Paszke et al., 2019) v1.9.0 and trained using the framework of monai (MONAI Consortium, 2021) v0.6.0. The MR image files were stored as Neuroimaging Informatics Technology Initiative (NIfTI) format, and processed using a Simple Insight Toolkit (SimpleITK; Lowekamp et al., 2013) v2.1.0. The visualized results were presented using ITK-SNAP (Yushkevich et al., 2006) v3.8.0.

### Results on the Test Set

**FIGURE 6:**
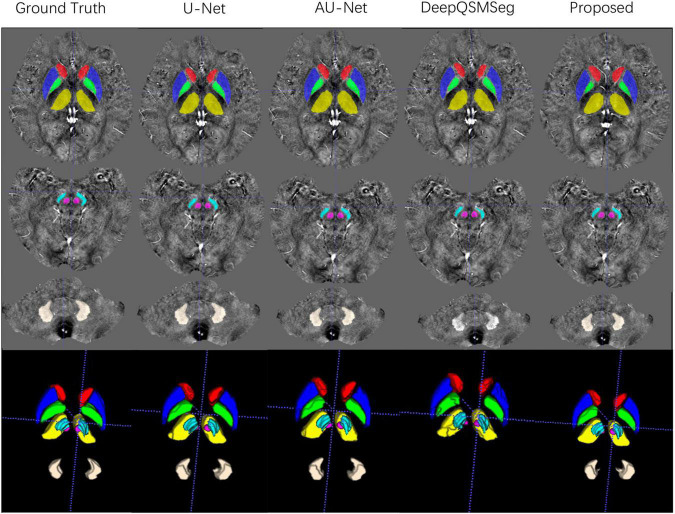
Visualized examples of the segmentations of manual delineation, U-Net, AU-Net, DeepQSMSeg, and our proposed method on the test set of Dataset I. The segmentation results were overlaid on QSM images.

We evaluated the segmentation performance on the test dataset with 20 subjects. Figure 6 presents the visualized examples on a randomly chosen subject. As we can see, U-Net, AU-Net, and our proposed CAU-Net achieved better performance than DeepQSMSeg. The DeepQSMSeg failed in identifying DNs on all subjects. To further compare the results, we presented the DC, SDC, ASSD and HD95 of our interested deep nuclei in Table 2. To compare the overall performance, the mean value of the metrics over all 7 nuclei was also presented in the last column.

**TABLE 2:** Numerical evaluation results on the test set.

Metric		CN	GP	PUT	THA	SN	RN	DN	Average
DC	U-Net	0.8232	0.8620	0.8582	0.8595	0.7142	0.8271	0.8050	0.8213
	AU-Net	0.8128	0.8472	0.8559	0.8563	0.7052	0.8418	0.**8056**	0.8178
	DeepQSMSeg	0.7551	0.8278	0.8137	0.7997	0.6391	0.7966	0	0.6617
	Proposed	**0.8306**	**0.8694**	**0.8629**	**0.8595**	**0.7161**	**0.8465**	0.7950	**0.8257**
SDC	U-Net	0.8169	0.8843	0.8596	0.76	0.8588	0.9354	0.8661	0.8544
	AU-Net	0.8033	0.8677	0.8584	0.7582	0.852	0.9431	**0.8701**	0.8504
	DeepQSMSeg	0.7167	0.8321	0.7968	0.6533	0.7864	0.911	0	0.6709
	Proposed	**0.8240**	**0.8913**	**0.8675**	**0.7648**	**0.8600**	**0.9515**	0.8549	**0.8591**
ASSD (mm)	U-Net	0.5183	0.3272	0.3881	0.5544	0.3794	0.2235	0.3614	0.3932
	AU-Net	0.5548	0.3725	0.3972	0.5620	0.3994	0.1934	**0.3540**	0.4047
	DeepQSMSeg	0.7622	0.4693	0.5448	0.8131	0.5503	0.2705	∞	/
	Proposed	**0.4995**	**0.3055**	**0.3676**	**0.5505**	**0.3763**	**0.1837**	0.3992	**0.3832**
HD95 (mm)	U-Net	2.7923	1.6541	1.9375	2.1780	2.1834	1.2474	**1.9552**	1.9926
	AU-Net	2.9685	1.8428	1.9730	2.1899	2.2985	1.1228	2.0141	2.0585
	DeepQSMSeg	3.5513	2.3229	2.5590	2.9516	2.9403	1.3858	∞	/
	Proposed	**2.6380**	**1.5405**	**1.8432**	**2.1323**	**2.0489**	**1.0143**	2.1697	**1.9124**

*The most prominent value for each metric is highlighted in bold font. DeepQSMSeg fails in segmenting DN on all subjects.*

As we can see from Table 2, our proposed CAU-Net achieved the best segmentation accuracy on all nuclei except DN. On DN, our proposed method was slightly worse than AU-Net. Across the nuclei, all methods achieved lower DC and SDC values on SN. The main reason was that the DC was more sensitive to small objects, while ASSD and HD95 were equally sensitive to small and large objects.

**FIGURE 7:**
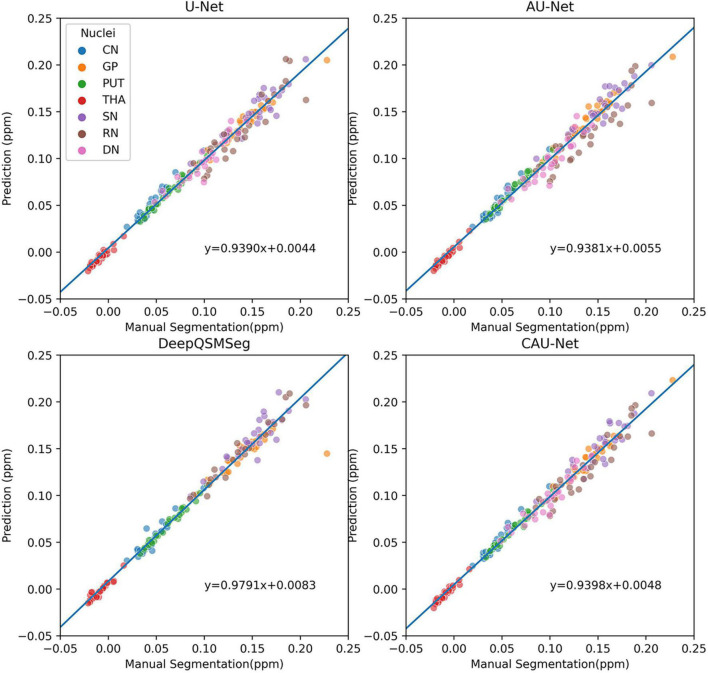
Scatter plot of susceptibility values measured from manual segmentations and automatic segmentations on the subjects of the test set of Dataset I. The correlation lines are also plotted. For DeepQSMSeg, we omitted the results on DN.

To further show the accuracy in segmenting each nucleus of each subject, we plotted scatter maps to show the correlations between the ground truth and the predictions in terms of the measured susceptibility values, as shown in Figure 7. Our proposed method presented the highest correlation with the manual delineations, while the DeepQSMSeg presented the lowest. As the DeepQSMSeg was originally developed on a large QSM dataset with 631 subjects, which is much larger than ours, it did not include as many data augmentation approaches as we did, and it may lead to the performance reduction compared with that reported by Guan et al. (2021).

### Results on Dataset II

**FIGURE 8:**
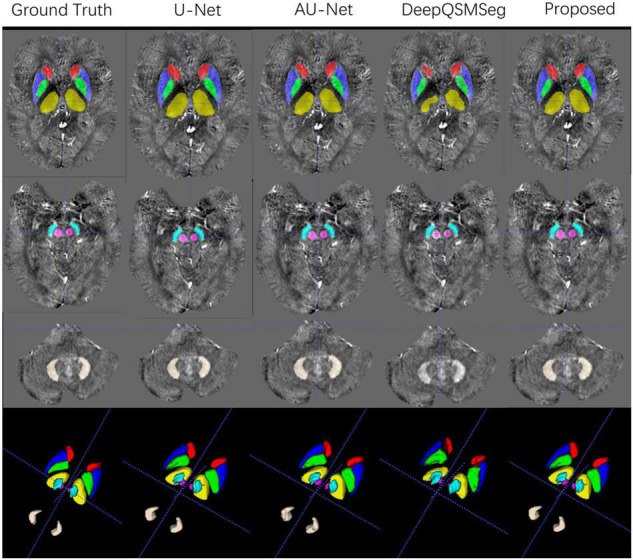
Visualized examples of the segmentations of manual delineation, U-Net, AU-Net, DeepQSMSeg, and our proposed method on Dataset II. The segmentation results were overlaid on QSM images.

All subjects in Dataset II were used as an additional test set. We adopted the networks trained on the training set of Dataset I to generate the segmentation maps on the subjects in Dataset II. Figure 8 presents some visualized examples. As we can see from Figure 8, most methods presented good segmentation accuracy on Dataset II. For DeepQSMSeg, it can still not segment the DN out, which implies that it may not be able to learn the features of DN.

To better investigate the segmentation accuracy, the segmentation metrics are summarized in Table 3. As we can see, our proposed method achieved the best performance in terms of mean DC, mean SDC, and mean ASSD. The U-Net performed the best on HD95, while our proposed CAU-Net had a very close performance. Our proposed method achieved the highest segmentation accuracy on CN, GP, PUT, and RN, while U-Net is the best on THA and DN.

**TABLE 3:** Numerical evaluation results on the Dataset II.

Metric		CN	GP	PUT	THA	SN	RN	DN	Average
DC	U-Net	0.7694	0.8428	0.8502	**0.7920**	0.6724	0.7685	**0.8472**	0.7918
	AU-Net	0.7511	0.8507	0.8517	0.7811	0.6949	0.7725	0.8381	0.7914
	DeepQSMSeg	0.7129	0.8199	0.8232	0.7516	**0.7544**	0.7145	0	0.6538
	Proposed	**0.7792**	**0.8519**	**0.8561**	0.7782	0.6816	**0.7750**	0.8448	**0.7953**
SDC	U-Net	0.7741	0.8729	0.8919	**0.6565**	0.8199	**0.9030**	**0.9138**	0.8331
	AU-Net	0.7565	0.8860	0.8942	0.6370	0.8343	0.8983	0.9044	0.8301
	DeepQSMSeg	0.7194	0.8491	0.8559	0.6054	**0.8942**	0.8800	0.0000	0.6863
	Proposed	**0.7847**	**0.8866**	**0.9005**	0.6343	0.8258	0.8994	0.9109	0.8346
ASSD (mm)	U-Net	0.6605	0.3863	0.3536	**0.9056**	0.5126	0.2995	**0.2683**	0.4838
	AU-Net	0.7164	**0.3482**	0.3445	0.9447	0.4704	0.3055	0.2924	0.4889
	DeepQSMSeg	0.8148	0.4584	0.4326	1.0112	**0.3216**	0.3625	∞	/
	Proposed	**0.6246**	0.3535	**0.3334**	0.9515	0.4958	**0.2992**	0.2764	**0.4763**
HD95 (mm)	U-Net	2.9219	1.9752	1.8634	**2.8873**	2.7109	**1.5878**	**1.6721**	**2.2312**
	AU-Net	3.1908	**1.9015**	1.9278	2.9552	2.5247	1.6501	1.7910	2.2773
	DeepQSMSeg	3.3003	2.3239	2.1374	3.2131	**1.9669**	1.7993	∞	/
	Proposed	**2.9194**	2.0064	**1.8305**	2.9671	2.5369	1.6748	1.7071	2.2346

As Dataset II was acquired by using a different machine with different parameters from the training set, the overall performance of all methods degraded compared to their performance on the test set of Dataset I. Interestingly, when segmenting DN, all methods had better accuracy on Dataset II, which implies that the QSMs stemmed from STAGE had better tissue contrast on DN.

**FIGURE 9:**
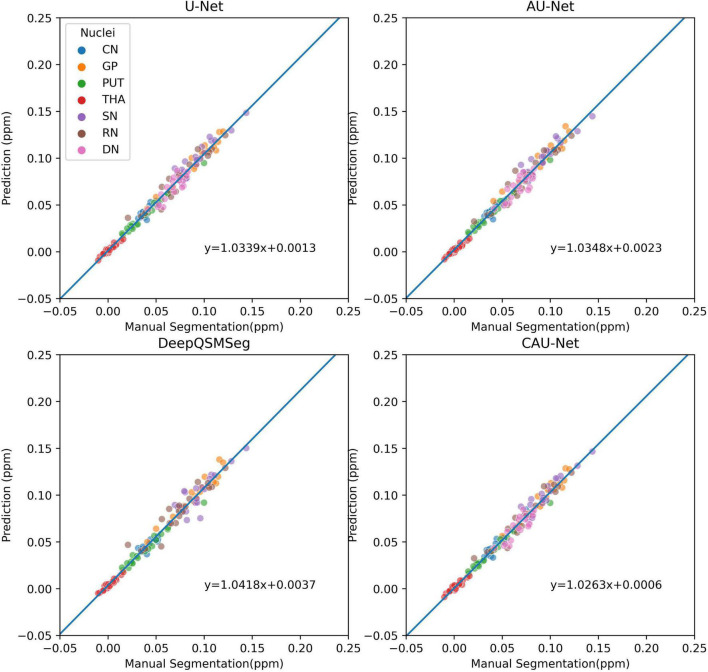
Scatter plot of susceptibility values measured from manual segmentations and automatic segmentations on the subjects of the Dataset II. The correlation lines are also plotted. For DeepQSMSeg, we omitted the results on DN.

The correlations of the measured susceptibility values between the manual segmentations and the automatic segmentations are also plotted in Figure 9. As shown in the figure, all segmentation methods presented a high agreement with the values measured on manual segmentations. DeepQSMSeg had the highest correlation, which, however, was calculated by omitting the DN. Our proposed method achieved the highest performance among the other three methods, which all successfully segmented all nuclei.

## Discussion

To further investigate the impact of various training and inference strategies on the segmentation accuracy, the segmentation performance under different training and inference settings is discussed.

### Test Time Augmentation

In our proposed method, we adopted TTA to improve the segmentation accuracy during inference. To illustrate the impact of TTA on the segmentation accuracy, we evaluated the segmentation performance without using TTA as summarized in Table 4. To clearly illustrate the gain, the column “Delta” explicitly quantized the improvement on the average metrics of all 7 gray matter nuclei. As we can see from Table 4, U-Net, AU-Net, and CAU-Net had achieved prominent improvement on the DCs of all nuclei. DeepQSMSeg, on the other hand, suffered from performance loss when TTA was introduced. It implied that the DeepQSMSeg might overfit on the training set. On the other hand, our proposed method presented the most significant difference with and without TTA. It implied that our proposed CA module might be able to filter irrelevant features from the encoder output feature maps, and had less risk in overfitting.

**TABLE 4:** Segmentation performance on the test set of Dataset I without adopting TTA during inference.

Metric		CN	GP	PUT	THA	SN	RN	DN	Average	Delta
DC	U-Net	0.8204	0.8594	0.8577	0.8593	0.7134	0.8215	0.8029	0.8192	−0.0021
	AU-Net	0.8112	0.8472	0.8533	0.852	0.7021	0.8421	0.7991	0.8153	−0.0025
	DeepQSMSeg	0.7943	0.8447	0.8348	0.8375	0.6669	0.8194	0	0.6854	+0.0237
	Proposed	0.8263	0.8669	0.8598	0.859	0.7097	0.8383	0.794	0.822	−0.0037
SDC	U-Net	0.8252	0.8897	0.8719	0.7765	0.8609	0.9342	0.8613	0.86	+0.0056
	AU-Net	0.8183	0.8753	0.87	0.7661	0.858	0.9453	0.8661	0.857	+0.0066
	DeepQSMSeg	0.77	0.8545	0.8299	0.7319	0.8098	0.9289	0	0.7036	+0.0327
	Proposed	0.8177	0.889	0.8641	0.7662	0.8562	0.9498	0.8528	0.8565	−0.0026
ASSD	U-Net	0.5045	0.3142	0.3646	0.5285	0.3736	0.2289	0.3708	0.3836	−0.0096
(mm)	AU-Net	0.519	0.3521	0.3681	0.5521	0.3872	0.1896	0.3651	0.3904	−0.0143
	DeepQSMSeg	0.6277	0.4268	0.4786	0.6453	0.4964	0.2361	∞	∞	/
	Proposed	0.5147	0.3133	0.3754	0.5514	0.3823	0.1957	0.4025	0.3908	+0.0076
HD95	U-Net	2.7117	1.5892	1.8551	2.108	2.0384	1.258	1.9874	1.9354	−0.0572
(mm)	AU-Net	2.7192	1.7431	1.8281	2.123	2.2067	1.1061	2.0545	1.9687	***−0.0898***
	DeepQSMSeg	3.1397	2.8627	2.2427	2.4159	2.4404	1.3513	∞	∞	/
	Proposed	2.6915	1.5994	1.8388	2.104	2.0481	1.1243	2.1912	1.9425	+0.0301

*The Delta value indicates the difference with and without TTA.*

As Table 4 shows, the TTA was an effective method for improving the DC. However, it is interesting to observe from Table 4 that when TTA is adopted, the performance of U-Net and AU-Net became worse in terms of surface distance metrics, i.e., SDC, ASSD, and HD95. Our proposed CAU-Net, on the other hand, presented substantial performance improvement in all metrics when TTA was adopted. Such a phenomenon indicates that the CAU-Net could be much more robust to the input variations, and the generalization ability of the proposed CAU-Net is stronger than that of other comparative methods. The improvement in the generalization capability should be attributed to the high-pass filter nature of the CA module. By filtering out unnecessary information and only preserving the edge information on each feature map, the feature maps fed to the decoder layers were simplified by the CA module, making the decoder layers easier to utilize the spatial information.

The expense of using TTA was, however, the inference time. With TTA, as we had to make predictions on the original and augmented images, we had to take much more time for inference. For instance, in our study, as we generated 9 augmented images, the inference time with TTA would be 10 times that without TTA.

### Training Strategy

This subsection would like to demonstrate the importance of properly designed training strategies. In our proposed method, we adopted data augmentation, deep supervision, and a nonuniform patch sampling strategy to train the neural network well. To demonstrate the effectiveness of training strategies, we trained the CAU-Net using different training setups. In particular, in the three experiments shown in Table 5, we removed the nonuniform patch sampling, deep supervision, and data augmentation as shown in Table 1 to see their contributions to the segmentation accuracies. To make it clear, we only presented the average values of DC, SDC, ASSD, and HD95. As we can see, the segmentation accuracies reduced in all the three additional experiments, indicating that they contributed to improving the segmentation performance. The data augmentations contributed most to the DC, while the deep supervision was the most essential factor in terms of surface distance metrics.

**TABLE 5:** Segmentation performance on the test set under different training strategies.

Method	DC	SDC	ASSD (mm)	HD95 (mm)
Proposed	0.8257	0.8591	0.3832	1.9124
w/o nonuniform patch sampling	0.8108 (−0.0149)	0.8455 (−0.0136)	0.4259 (+0.0427)	2.1384 (+0.2260)
w/o deep supervision	0.8048 (−0.0209)	0.8324 (−0.0267)	0.4500 (+0.0668)	2.2460 (+0.3336)
w/o data augmentation	0.7893 (−0.0364)	0.8461 (−0.0130)	0.4242 (+0.0410)	2.1782 (+0.2658)

In particular, data augmentation techniques have been shown to be one of the most essential approaches in image segmentations. It has been well known that data augmentation approaches have been beneficial for improving the classifiers’ performance since the success of AlexNet. In our study, we used various data augmentation methods to improve the performance as summarized in Table 1. As shown in Table 5, after removing the data augmentations from training, the DC significantly drops from 0.8257 to 0.7893. The main reason should be blamed for the small size of our dataset. When data augmentation was adopted, the methods listed in Table 1 can generate many different versions of images, which increased the diversity of the training data and improved the representation ability of the neural network.

Deep supervision was also a critical approach that affected the performance. As Table 5 shows, when deep supervision is absent, the DC drops to 0.0209 and the SDC drops to 0.0267. In our study, deep supervision is implemented by adding a convolution layer at each decoder stage to generate an auxiliary segmentation map. By incorporating additional classifier outputs at the middle stages, the network can be forced to learn effective representations to reduce the loss. Moreover, it also helped the deeper layers to be updated at the beginning of training, and was beneficial for improving the convergence behavior. By introducing deep supervision at the decoder layers, all the decoder layers were forced to extract spatial information from the skip connections. Combined with the edge information extracted from the CA module, much finer segmentation maps can be obtained as the decoder layers recover the feature maps to their original resolution.

The effect of the patch sampling scheme was also discussed. In our task, as it is not possible to feed the whole volume into the memory due to the limited memory size, splitting the images into patches was necessary. In our study, we chose to sample the patches with bias because the foreground voxels (i.e., the nuclei) and the background voxels are severely imbalanced. In particular, the sampling method ensured that at least two-third patches were centered at the foreground voxel during training. The nonuniform patch sampling method can also be regarded as an implicit way of data augmentation, which over-samples the foreground voxels to train the network. As we can see from Table 5, the segmentation performance was slightly improved by using the nonuniform patch sampling scheme. Despite that the contribution is relatively small compared to the contributions of deep supervision and data augmentation, the nonuniform patch sampling was shown to be able to further improve the performance with almost no additional computational cost.

After all, to improve the segmentation accuracy, it has been shown in our experiments that the training strategy was at least as important as developing more advanced networks. In our study, we can see that the performance gain of our proposed method came from several aspects, which are as follows: (1) the CA module that reduces the redundant information passed to the decoder layers; (2) the deep supervision’s assistance in forcing the decoder layers to learn effective representations; (3) sufficient data augmentation and the bias patch sampling strategy to increase the diversity of patches; (4) TTA to utilize the ensembling of various predictions.

## Conclusion

In our study, a deep-learning-based method was proposed for the gray matter nuclei segmentation task on T_1_WI and QSM. A CA module was proposed and incorporated in the skip connections of U-Net to filter out the redundant information from the encoder feature maps. Experimental results on two test sets acquired with various parameters revealed that our proposed method could overperform all popular network structures. To investigate the origination of our proposed method, the results obtained under different training and inference strategies were also discussed, which implied that the appropriate choices of training and inference strategies were at least as important as developing more effective network structures.

## Data Availability Statement

The original contributions presented in the study are included in the article/supplementary material, further inquiries can be directed to the corresponding authors.

## Ethics Statement

The studies involving human participants were reviewed and approved by the Tianjin First Central Hospital Review Board and Ethics Committee. The patients/participants provided their written informed consent to participate in this study.

## Author Contributions

CC and ZL wrote this manuscript. MW and ZL implemented the programming work. CC and HW contributed to the data acquisition and data analysis. YC, SZ, and KZ contributed to the data evaluation and acquisition. WS and SX contributed to the study conception and design. ZL and SX contributed to the study conception and design, drafted and revised the article to provide important intellectual content, and agreed to be accountable for all aspects of this study. All authors contributed to the article and approved the submitted version.

## Conflict of Interest

The authors declare that the research was conducted in the absence of any commercial or financial relationships that could be construed as a potential conflict of interest.

## Publisher’s Note

All claims expressed in this article are solely those of the authors and do not necessarily represent those of their affiliated organizations, or those of the publisher, the editors and the reviewers. Any product that may be evaluated in this article, or claim that may be made by its manufacturer, is not guaranteed or endorsed by the publisher.
